# Extracellular Spermine Triggers a Rapid Intracellular Phosphatidic Acid Response in Arabidopsis, Involving PLDδ Activation and Stimulating Ion Flux

**DOI:** 10.3389/fpls.2019.00601

**Published:** 2019-05-21

**Authors:** Xavier Zarza, Lana Shabala, Miki Fujita, Sergey Shabala, Michel A. Haring, Antonio F. Tiburcio, Teun Munnik

**Affiliations:** ^1^Plant Cell Biology, Swammerdam Institute for Life Sciences, University of Amsterdam, Amsterdam, Netherlands; ^2^Plant Physiology, Swammerdam Institute for Life Sciences, University of Amsterdam, Amsterdam, Netherlands; ^3^Tasmanian Institute of Agriculture, University of Tasmania, Hobart, TAS, Australia; ^4^Gene Discovery Research Group, RIKEN Plant Science Center, Tsukuba, Japan; ^5^Department of Biology, Healthcare and the Environment, Faculty of Pharmacy, University of Barcelona, Barcelona, Spain

**Keywords:** phospholipase D (PLD), phosphatidic acid (PA), lipid signaling, polyamines (putrescine, spermidine, spermine), phospholipids, MIFE

## Abstract

Polyamines, such as putrescine (Put), spermidine (Spd), and spermine (Spm), are low-molecular-weight polycationic molecules found in all living organisms. Despite the fact that they have been implicated in various important developmental and adaptative processes, their mode of action is still largely unclear. Here, we report that Put, Spd, and Spm trigger a rapid increase in the signaling lipid, phosphatidic acid (PA) in Arabidopsis seedlings but also mature leaves. Using time-course and dose-response experiments, Spm was found to be the most effective; promoting PA responses at physiological (low μM) concentrations. In seedlings, the increase of PA occurred mainly in the root and partly involved the plasma membrane polyamine-uptake transporter (PUT), RMV1. Using a differential ^32^P_i_-labeling strategy combined with transphosphatidylation assays and T-DNA insertion mutants, we found that phospholipase D (PLD), and in particular *PLDδ* was the main contributor of the increase in PA. Measuring non-invasive ion fluxes (MIFE) across the root plasma membrane of wild type and *pldδ-*mutant seedlings, revealed that the formation of PA is linked to a gradual- and transient efflux of K^+^. Potential mechanisms of how *PLDδ* and the increase of PA are involved in polyamine function is discussed.

## Introduction

Polyamines are small polycationic molecules present in all living organisms (Galston and Kaur-Sawhney, 1995). In plants, putrescine (Put), spermidine (Spd), and spermine (Spm) are the major polyamines, where they have been implicated in a broad range of cellular events, including embryogenesis, cell division, morphogenesis, senescence, and in various biotic- and abiotic stress responses (Bagni and Pistocchi, 1988; Wallace, 2009; Tiburcio et al., 2014; Michael, 2016). Despite the fact that polyamines were discovered nearly 350 years ago, and have been intensively studied during the last decades, the molecular mechanism by which these molecules regulate such a wide range of cellular functions remains a big mystery (Bachrach, 2010; Alcázar and Tiburcio, 2014). Nonetheless, polyamines have been shown to interact with components of the nucleus and cellular membranes, including transcription factors, protein kinases and phospholipases (Miller-Fleming et al., 2015) as well as ion transporting proteins (Pottosin and Shabala, 2014; Pottosin et al., 2014). The multifaceted relationship between polyamine-mediated effects and the activation of different signaling systems adds another layer of complexity to the experimental determination of direct polyamine targets (Alcázar et al., 2010; Tiburcio et al., 2014; Miller-Fleming et al., 2015).

While most studies have focused on the interaction of endogenous polyamines with immediate subcellular targets, plants are also exposed to extracellular polyamines. In the soil, plants encounter a high degree of polyamines through decomposition of organic material by microorganisms (Young and Chen, 1997; Zandonadi et al., 2013). In addition, there are several environmental cues, such as salinity stress and abscisic acid (ABA), which trigger an efflux of polyamines into the apoplast (Moschou et al., 2008; Toumi et al., 2010). There, polyamines can be oxidized by diamine- and polyamine oxidases, producing H_2_O_2_ that in turn triggers downstream effects that eventually affect the plant’s development and/or responses to stress (Takahashi et al., 2003; Moschou et al., 2008; Toumi et al., 2010; Pottosin and Shabala, 2014). However, not all apoplastic polyamines are oxidized, as intercellular transport and local internalization of a substantial part of these compounds also takes place (Friedman et al., 1986; Pistocchi et al., 1987; Ditomaso et al., 1992a; Yokota et al., 1994; Sood and Nagar, 2005; Pommerrenig et al., 2011).

The study of polyamine uptake and transport in plant cells remains scarce. However, with the recent characterization of several polyamine-uptake transporters (PUTs), an important new area is emerging, providing interesting genetic tools to explore its potential in plant function and signaling (Fujita et al., 2012; Mulangi et al., 2012; Li et al., 2013; Strohm et al., 2015; Martinis et al., 2016; Tong et al., 2016).

Phosphatidic acid (PA) represents a minor class of membrane lipids, constituting 1–3% of total phospholipids in most plant tissues. As a precursor of glycerolipids, PA is involved in lipid biosynthesis at the ER and plastids. Over the last decade, however, PA has also emerged as a signaling molecule, playing key roles in regulating plant development and stress responses (Munnik, 2001; Testerink and Munnik, 2005, 2011; Yao and Xue, 2018). This PA is typically formed at the plasma membrane and along the endosomal membrane system, where it recruits and modulates target proteins involved in membrane trafficking, organization of the cytoskeleton and ion transport (Testerink and Munnik, 2005, 2011; Kooijman et al., 2007; Raghu et al., 2009; McLoughlin et al., 2013; Pleskot et al., 2013; Putta et al., 2016; Yao and Xue, 2018). A local increase of PA may also induce biophysical effects, affecting membrane curvature and surface charge, which facilitate membrane fission and fusion (Kooijman et al., 2003; Wang et al., 2006; Roth, 2008), also in cooperation with other lipid signals (Testerink and Munnik, 2011).

The accumulation of PA in response to stimuli is in general relatively fast, taking place within minutes after stimulation, and is generated via two pathways, i.e., via phosphorylation of diacylglycerol (DAG) by DAG kinase (DGK) and by hydrolysis of structural phospholipids by phospholipase D (PLD). DAG itself can be produced via non-specific phospholipase C (NPC), which hydrolyses structural phospholipids, or by phosphoinositide- (PI-) specific phospholipase C (PLC), which hydrolyses inositol-containing phospholipids (Munnik, 2014).

Both PLC- and PLD activities are known to be affected by polyamines. *In vitro* studies on isolated enzymes from animal cells and tissues have shown that polyamines can inhibit (Kimura et al., 1986; Smith and Snyderman, 1988; Wojcikiewicz and Fain, 1988; Sjöholm et al., 1993; Pawelczyk and Matecki, 1998) and stimulate PLC activity (Sagawa et al., 1983; Haber et al., 1991; Späth et al., 1991; Periyasamy et al., 1994; Pawelczyk and Lowenstein, 1997) and PLD activity (Jurkowska et al., 1997; Madesh and Balasubramanian, 1997). In plants, polyamines have been found to activate PLC in *Catharanthus roseus* roots (Echevarría-Machado et al., 2002, 2004), but to inhibit it in *Coffea arabica* cells, where an increase in PLD activity was observed (Echevarría-Machado et al., 2005).

Here, we show that polyamines trigger a rapid (minutes) PA response in Arabidopsis seedlings, with Spm being the most potent. Using differential ^32^P_i_-labeling techniques and a PLD-specific transphosphatidylation assay (Arisz and Munnik, 2013; Munnik and Laxalt, 2013), we provide evidence that the PLD pathway is the most important contributor. Using T-DNA-insertion PLD mutants, we identified PLDδ as the main contributor of the Spm induced-PA response. Using Microelectrode Ion Flux Estimation (MIFE), we found a differential Spm induced-K^+^ efflux response in the pldδ KO mutant, highlighting a potential role for PA downstream of Spm signaling.

## Materials and Methods

### Plant Material and Growth Conditions

*Arabidopsis thaliana pldα1, pldα3, pldδ, pldε, pldα1/δ, pldα1/δ/α3, pldα1/δ/ε, pldζ1, pldζ2, gapc1-1/gapc2-1, gapc1-1/gapc2-2*, *rmv1, spms-2* mutant null alleles and the *Pro35S::RMV1 and Pro35S::SPMS-9* transgenic lines were described previously (Hong et al., 2008, 2009; Bargmann et al., 2009; Gonzalez et al., 2011; Fujita et al., 2012; Guo et al., 2012; Galvan-Ampudia et al., 2013). The *lat1/2/3/5* and *lat1/2/4/5* quadruple null mutant were generated by Dr. M. Fujita (unpublished; RIKEN Plant Science Center, Japan), while *pldα1/δ/α3* and *pldα1/δ/ε* triple knock-out mutants were kindly provided by Prof. Dr. D. Bartels (University of Bonn, Germany). In most cases *Arabidopsis thaliana* ecotype *Col-0* was used as wild type, except for *rmv1* and the *Pro35S::RMV1* lines, in which *Ler* ecotype and Col-0 empty vector, *Ve-1*, were used as wild type, respectively.

Seeds were surface-sterilized with chlorine gas and sown under sterile conditions on square petri dishes containing standard growth medium consisting of ½ Murashige and Skoog (MS) medium with Gamborg B5 vitamins (pH 5.7; KOH), 1% (w/v) sucrose, and 1% (w/v) agar. Plates were vernalized at 4°C for 48 h and then placed vertically under the angle of 70°, in a growth chamber (16/8 light/dark cycle, 110–130 μmol m^–2^ s^–1^) at 22°C. Five days-old seedlings were then transferred to either 2 mL Eppendorf safe-lock tubes for ^32^P_i_ labeling O/N, or to treatment plates for phenotypic analyses.

### Chemicals

All chemicals were obtained from Sigma-Aldrich except ^32^P_i_ (orthophosphate, ^32^PO_4_^3–^), which was purchased from PerkinElmer. Arabidopsis incubations with polyamines and chemicals were performed in incubation buffer, consisting of 2.5 mM MES buffer [2-(N-morpholino) ethanesulfuric acid], pH 5.7 (KOH), 1 mM KCl.

### ^32^P_i_-Phospholipid Labeling, Extraction and Analysis

Phospholipid levels were measured as described earlier (Munnik and Zarza, 2013). Briefly, three seedlings per sample were metabolically labeled overnight by flotation in continuous light in 2 ml safe-lock Eppendorf tubes containing 200 μl incubation buffer (2.5 mM MES-KOH, pH 5.7, 1 mM KCl) and 2.5–10 μCi ^32^PO_4_^3–^ (stock ^32^P_i_; carrier-free, 2.5–10 μCi/μL). For mature plants, Arabidopsis leaf disks (∅ 5 mm) were taken from 3-week-old plants and labeled using the same conditions. Treatments were performed by adding 1:1 (v/v) of a 2× solution and incubations were stopped at indicated times by adding perchloric acid (Munnik and Zarza, 2013). Lipids were extracted and analyzed by thin-layer chromatography (TLC) using an ethyl acetate solvent system (Munnik and Laxalt, 2013). Radioactivity was visualized by autoradiography and individual spots were quantified by phosphoimaging (Typhoon FLA 7000; GE Healthcare).

For certain experiments, the protocol was slightly modified, i.e.: (1) In short-labeling experiments, ^32^Pi was added 30 min prior treatment. (2) In transphosphatidylation assays, treatments were performed in the presence of 0.5% *n*-butanol (Munnik and Laxalt, 2013). (3) For tissue-dissection experiments, seedlings were labeled, treated and fixed as described above, but then carefully cut into sections with a scalpel, and every section processed separately.

For statistical analysis, letters indicate values significantly different according to Student–Newman–Keuls test at *P*-value <0.05, and asterisks indicate significant differences with respect to control treatments, using the Student’s *t*-test: *P < 0.05, **P < 0.01, ***P < 0.005. Data represent the mean ± SD. The results obtained were confirmed by at least 2 independent experiments.

### Detection of ROS and NO in Arabidopsis Root

ROS production in the root tip of 5-day-old seedlings was detected by DCF fluorescence as described previously (Pei et al., 2000; Zhang et al., 2009). Briefly, seedlings were treated for the indicated times and then transferred to 10 μM H_2_DCFDA for 10 min followed by two washes in buffer. For ROS scavenging, seedlings were pre-treated with 5 mM N,N’-Dimethylthiourea (DMTU; Lu et al., 2009) for 60 min, before the different treatments with or without Spm 120 μM for 30 min. For NO detection, seedlings were co-incubated with the corresponding treatment and 10 μM DAR-4M for 30 min, and then washed two times with buffer. For cPTIO treatment, 0.1 mM cPTIO was applied for 60 min prior to treatments in order to scavenge NO. All incubations were performed in dark conditions. The localization of the DCF and DAR signal was done using the AMG Evos FL digital inverted microscope equipped with transmitted light GFP (470/22 to 510/42 nm). Images were converted to 8-bit using Image-J, and data was quantified as mean pixel intensity per region of interest (ROI).

### Ion Flux Measurement

Net K^+^ fluxes were measured using MIFE technique (UTas Innovation, Hobart, TAS, Australia) (Newman, 2001; Shabala et al., 2006). Five days-old Arabidopsis seedlings were placed into a 30 mL measuring chamber, containing 0.5 mM KCl, 0.2 mM CaCl_2_, 5 mM MES, 2 mM Tris base; pH 6.0. Roots were immobilized in a horizontal position (Bose et al., 2014) and preincubated in the above buffer for at least 30 min. Electrodes were positioned near the root surface at the elongation zone (less than 2 mm from the root cap junction). First, steady-state ion fluxes were recorded over a period of 5 min, after which different concentrations of Spm were applied and net ion fluxes measured.

### Root Phenotyping Assay on Plates

*Arabidopsis* seedlings were grown on vertical plates containing standard growth medium for 5 days. Then, seedlings were transferred to plates supplemented with or without Spm. Plates were scanned at indicated days after transfer (DAT) using an Epson Perfection V700 Scanner at 300 dpi. For root measurements EZ-Rhizo software was used (Armengaud et al., 2009). Main root (MR) growth was expressed as growth ratio (MR length divided by MR length at 0 DAT). A paired *t*-test in SPSS was used for statistical analysis.

## Results

### Polyamines Trigger the Formation of PA in Arabidopsis Seedlings

**FIGURE 1:**
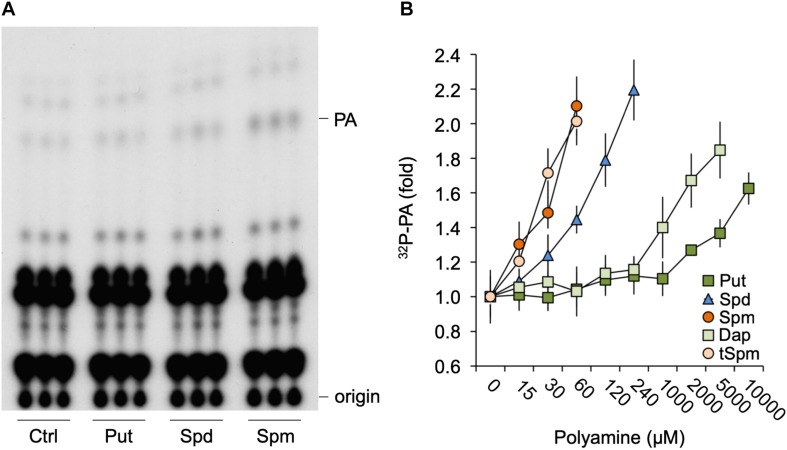
Polyamines trigger a PA response in *Arabidopsis* seedlings. **(A)** Six-days old seedlings that had been ^32^Pi-labeled O/N were treated for 30 min with 60 μM of putrescine (*Put*), spermidine (*Spd*) or spermine (*Spm*), or with buffer alone (control, *Ctrl*), after which lipids were extracted, separated by TLC, and visualized by autoradiography. Each sample represents the extract of three seedlings. **(B)** ^32^P-labeled PA fold response to 30 min Put, Spd, Spm or diaminopropane (*Dap*), and thermospermine (*tSpm*) at the indicated concentrations.

To investigate whether polyamines could affect PA signaling, Arabidopsis seedlings were ^32^P_i_-prelabeled O/N and treated with physiological concentrations (60 μM) of Put, Spd, or Spm for 30 min. As shown in Figure 1A, in particular Spm but also Spd, was found to induce an increase in PA. Put did not show any effect until millimolar concentrations were used (Figure 1B). Spm already induced significant PA responses at ≥15 μM, while 30–60 μM was required for Spd. Thermospermine (tSpm), a minor structural isomer of Spm also present in Arabidopsis (Kakehi et al., 2008), was found to induce PA responses at a similar concentrations as Spm (Figure 1B). Diaminopropane (Dap), a diamine product of polyamine oxidation, exhibited a similar low potency like Put (Figure 1B). Together these results show that polyamines can trigger a PA response, with its potency depending on charge, i.e., Spm^4+^ = tSpm^4+^ > Spd^3+^>>Put^2+^≈Dap^2+^.

**FIGURE 2:**
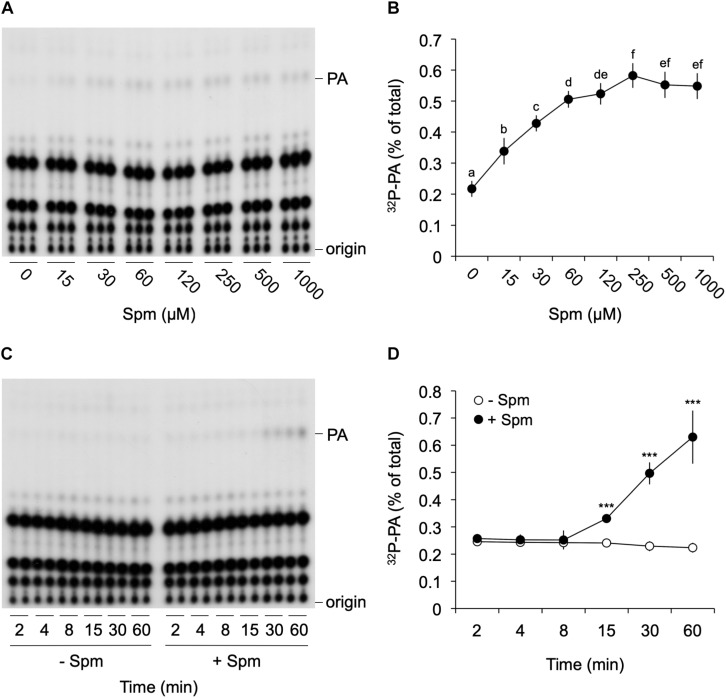
Dose-response and time-course analysis of the Spm induced-PA response. Six-days old ^32^P_i_-prelabeled seedlings were treated for 30 min with different concentrations of Spm **(A,B)** or with 60 μM Spm or buffer alone (control) for different times **(C,D)** and their lipids analyzed. **(A,C)** Autoradiographs of a representative experiment. **(B,D)** Quantifications of the PA response, showing the percentage of ^32^P-PA with respect to the total amount of ^32^P-labeled phospholipids. Data are the mean ± SD of three independent experiments (*n* = 9 for **B**; *n* = 6 for **D**). Different letters indicate values significantly different according to Student-Newman-Keuls test at *P*-value < 0.05. Asterisks indicate significant differences with respect to control treatments, using the Student’s *t*-test: *P < 0.05, ^∗∗^*P* < 0.01, ^∗∗∗^*P* < 0.005.

To further investigate the Spm-induced PA, detailed dose-response and time-course analyses were performed. As shown in Figure 2, Spm induced a clear dose-dependent Michaelis–Menten PA curve when treated for 30 min; starting at low μM levels and reaching a maximum 2.5-fold increase at ∼250 μM (Figures 2A,B). The response was relatively fast, starting between 8 and 15 min when using 60 μM of Spm, and PA linearly increasing (Figures 2C,D).

### Spm Triggers PA Formation in Roots and Requires Transport Across the Plasma Membrane via RMV1

**FIGURE 3:**
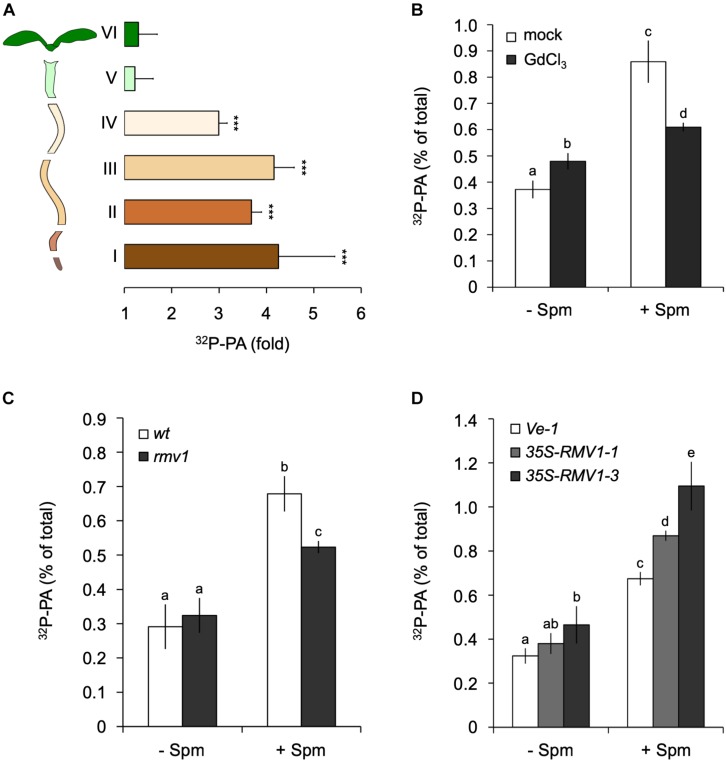
Spermine (Spm) induces PA responses in the root and involves polyamine-uptake transporter, RMV1. **(A)** ^32^P-PA response in different sections of ^32^P_i_-prelabeled Arabidopsis seedlings. Length/type of section: root tip, 2 mm (I), 3 mm (II), 5 mm (III), 5–7 mm (IV), hypocotyl (V), and cotyledons (VI). Results are expressed as fold-increase of PA with respect to control treatment of each section. **(B)** Gadolinium blocks Spm induced-PA response. O/N ^32^P-labeled seedlings were pre-treated with buffer containing KCl (mock) or 100 μM GdCl_3_ for 60 min after which they were treated with or without 60 μM Spm for 30 min. **(C,D)** ^32^P-PA responses in *rmv1* knock-out and two independent *Pro35S::RMV1* over-expressor lines treated for 30 min ± 60 μM Spm. Wild-type Landsberg (*Ler*) and a wild-type *Col-0* line containing the empty vector (*Ve-1*) were used as control lines, respectively. Different letters indicate values significantly different according to Student-Newman-Keuls test at *P*-value < 0.05. Asterisks indicate significant differences with respect to control treatments, using the Student’s *t*-test: *P < 0.05, ^∗∗^*P* < 0.01, ^∗∗∗^*P* < 0.005.

To obtain more information as to where in the seedling the PA accumulation took place, we performed the same ^32^P_i_-labeling and treatments, but now dissected root- and shoot tissues prior to lipid extraction. Interestingly, the Spm induced-PA increase was only found in the root, not in the shoot or hypocotyl (Figure 3A). Within the root, the PA accumulation was equally distributed along the different root sections (Figure 3A). A repetition of this experiment with 120 μM Spm gave similar results (data not shown). In contrast to seedlings, we did observe a Spm induced-PA response in ^32^P_i_-labeled leaf disks of mature, 3-weeks old plants (Supplementary Figure S1).

The non-permeant cation transport blocker, gadolinium (Gd^3+^) is known to inhibit the uptake of Spm across the plasma membrane (Pistocchi et al., 1988; Ditomaso et al., 1992b; Pottosin et al., 2014). Incubation of seedlings with GdCl_3_ prior to the application of Spm triggered a small PA response itself, but significantly reduced the Spm induced-PA response to approximately 70% of the control response (Figure 3B). This may indicate that most of the PA response observed is caused intracellularly.

To further characterize Spm uptake, we analyzed the *Arabidopsis* PUT/L-type amino acid transporter (LAT), called Resistant to Methyl Viologen 1 (RMV1, PUT3, LAT1), which is localized in the plasma membrane and responsible for the high-affinity uptake of Spm (Fujita et al., 2012). Using the knock-out T-DNA insertion mutant *rmv1* and two independent over-expressing *Pro35S::RMV1* lines, we found a 35% decrease and ∼20–40% increase in PA, respectively (Figures 3C,D). These results indicate that cellular uptake of Spm is required for the PA response, and that RMV1 is one of the proteins involved in internalizing Spm across the plasma membrane.

To functionally analyze the involvement of the rest of the PUT/LAT family members, of which Arabidopsis contains five homologs (Mulangi et al., 2012), two quadruple knock-out mutants were used, i.e., *lat1/2/3/5* and *lat1/2/4/5*, because the quintuple mutant was lethal (Fujita M., unpublished). Both lines, however, showed Spm induced-PA responses similar to wild type (Supplementary Figure S2). This discrepancy could be due to the fact that the single *rmv1*-KO allele is different from the quadruple mutants and belongs to a different ecotype (i.e., *Ler* vs. *Col-0*, respectively). While at least three LAT proteins exhibit polyamine transport activity (i.e., LAT1, LAT3, LAT4; Fujita et al., 2012; Mulangi et al., 2012), only LAT1 (RMV1, PUT3) is localized to the plasma membrane; LAT3 and LAT4 are localized to the ER and Golgi, respectively (Li et al., 2013; Fujita and Shinozaki, 2014). Hence, the results obtained may reflect distinct plasma membrane activity as well as genetic redundancy for Spm uptake, involving other members from the amino acid-polyamine-choline (APC) transporter family to which LAT/PUT transporters belong (Verrey et al., 2004; Rentsch et al., 2007).

### Spm-Triggered PA Is Predominantly, but Not Solely Generated via PLD

**FIGURE 4:**
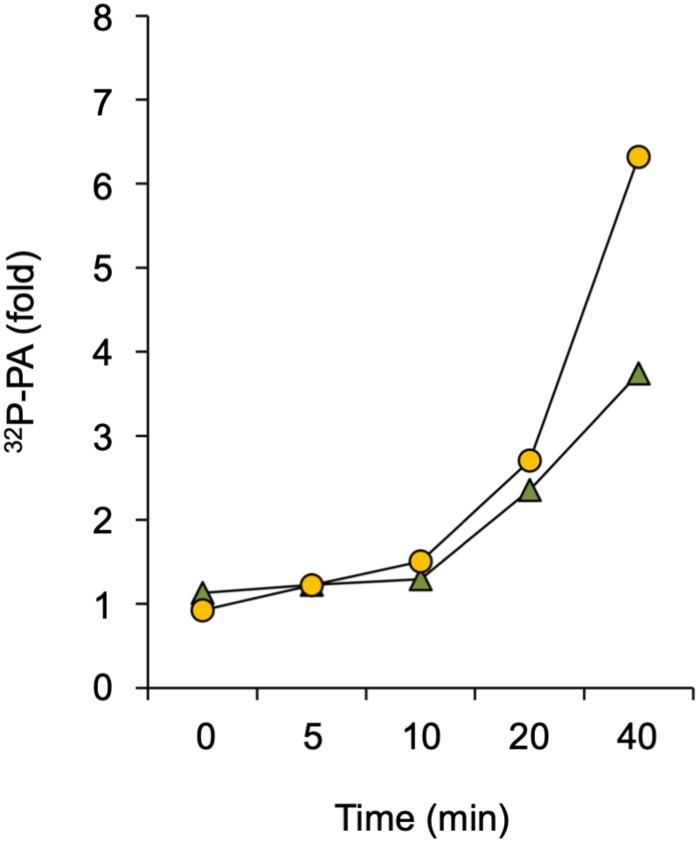
Analysis of DGK involvement in Spm triggered-PA response. Seedlings were pulse-labeled with ^32^P_i_ for 30 min and then treated with 60 μM Spm or buffer alone (Ctrl) for the indicated times. The fold-PA response of two independent experiments is shown (squares and triangles, respectively). Values were normalized to the ^32^P-labeling of phosphatidylinositol and to Ctrl, without Spm.

A rapid PA response has traditionally been associated with increased DGK- and/or PLD activity. DGK produces PA through phosphorylation of DAG that originates from the hydrolysis of phosphoinositides or structural phospholipids by PLC or NPC, respectively (Munnik, 2014). PLD hydrolyses structural phospholipids, like PE and PC, to form PA directly. To distinguish between these two routes, a differential ^32^P-labeling protocol was used that highlights the DGK kinase-dependent reaction (Arisz and Munnik, 2013). This method is based on the premise that the ^32^P_i_ added to seedlings is rapidly taken-up and incorporated into ATP and subsequently into lipids that are synthesized via kinase activity (e.g., DGK), which is in huge contrast to the relatively slow incorporation of ^32^P into structural phospholipids via *de novo* synthesis (Munnik et al., 1994, 1998; Arisz and Munnik, 2013). Under short labeling conditions, PLD would hardly generate ^32^P-labeled PA whereas the contribution of DGK would be augmented. As shown in Figure 4, Spm was still able to trigger an increase in ^32^P-PA when seedlings were only prelabeled for 30 min rather than 16 hrs O/N, indicating that at least part of the Spm induced-PA response is generated via DGK.

**FIGURE 5:**
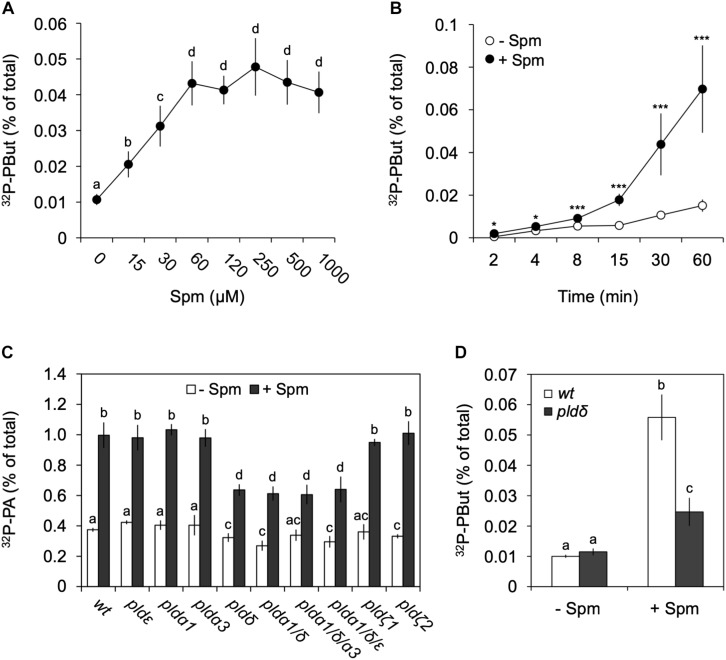
Analysis of Spm-induced PLD activity and gene identification. Dose-response **(A)** and time-course **(B)** experiments were performed as described earlier but this time in the presence of 0.5% *n*-butanol, to quantify the production of the PLD-dependent transphosphatidylation product, ^32^P-PBut. **(A,B)** Quantification of ^32^P-PBut. Data are the means ± SD of three independent experiments (*n* = 6–9). **(C)** ^32^P-PA responses in PLD T-DNA insertion mutants treated with (+) or without (–) 60 μM Spm for 30 min. Mutants analyzed: *plda1, plda3, pldδ, pldε, pldζ1, pldζ2*, and the mutant combinations *plda1/δ, plda1/δ/a3* and *plda1/δ/ε*. **(D)** ^32^P-PBut response in *pldδ* with respect to wt. Different letters indicate values significantly different according to Student-Newman-Keuls test at *P*-value < 0.05. Asterisks indicate significant differences with respect to control treatments, using the Student’s *t*-test: *P < 0.05, ^∗∗^*P* < 0.01, ^∗∗∗^*P* < 0.005.

To analyze the potential involvement of PLD, its unique ability to catalyze a transphosphatidylation reaction was used, which produces phosphatidylbutanol (PBut) *in vivo* in the presence of a low concentration of *n*-butanol (Munnik et al., 1995, 1998; Arisz and Munnik, 2013; Munnik and Laxalt, 2013). To get a substantial proportion (though not all) of the structural phospholipids (i.e., PLD’s substrate) ^32^P-labeled, seedlings were incubated with ^32^P_i_ O/N and the next day treated with Spm in the presence of 0.5% *n-*butanol. The subsequent formation of ^32^P-PBut is an *in vivo* marker for PLD activity that can be quantified. Again dose-response and time-course experiments were performed, but this time in the presence of *n*-butanol. As shown in Figures 5A,B, Spm clearly triggered PLD activity, with the PBut following a similar pattern as PA (Figure 2).

Arabidopsis contains 12 *PLDs*, i.e., 3 *PLDαs*, *2 PLDβs*, *3 PLDγs*, 1 *PLDδ*, 1 *PLDε*, and 2 *PLDζs* (Zhang et al., 2005). Validating the ^32^P-labeled PBut- and PA response in various T-DNA KO mutants, we identified PLDδ as the main contributor, with the *pldδ*-KO mutant alone or in combination with other KOs, showing a ∼55% reduction in PA and ∼70% reduction in PBut accumulation (Figures 5C,D). Interestingly, in Arabidopsis this isoform is located in the plasma membrane (Wang and Wang, 2001; Pinosa et al., 2013), while promoter-GUS analyses suggests it is mainly expressed in roots (Katagiri et al., 2001), which is in agreement with the PA response observed here.

### H_2_O_2_ or NO Are Not Involved in the Spm Induced-PA Response

**FIGURE 6:**
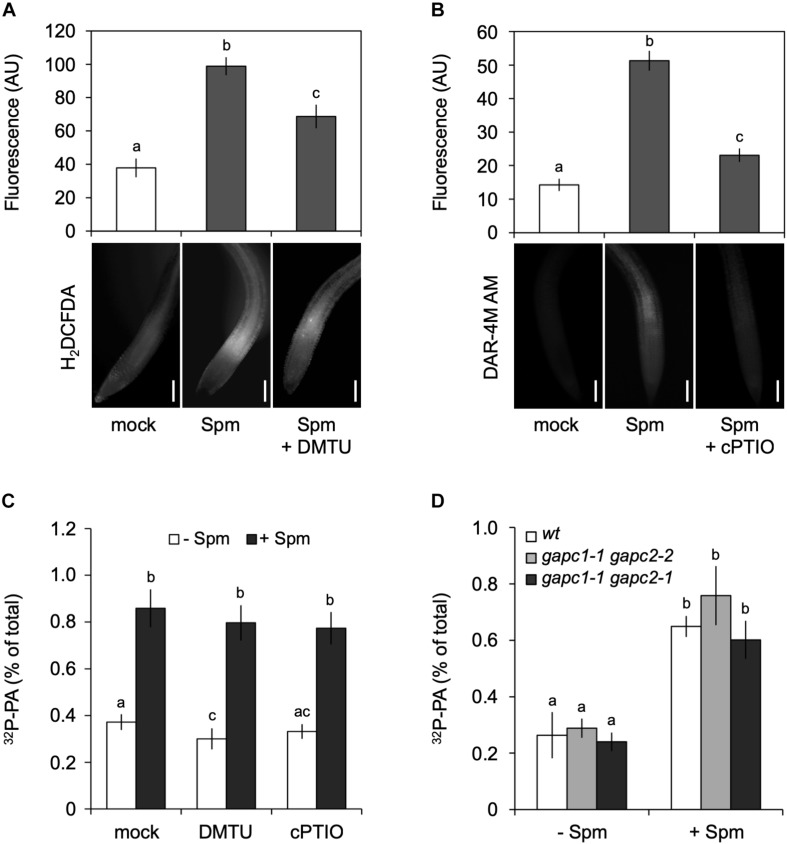
Spermine (Spm) induced-PA response is not generated through the production of H_2_O_2_ or NO. Visualization and quantification of Spm induced-H_2_O_2_ **(A)** or -NO production **(B)** using corresponding dyes and scavengers, respectively (H_2_DCFDA and DMTU for H_2_O_2_; DAR-4M AM and cPTIO for NO). Bars represent 100 μm. The fluorescence in the root tip is expressed as arbitrary units (AU; Mean ± SD, *n* = 10). **(C)** ^32^P-PA levels in seedlings incubated in presence of buffer (Ctrl) or scavengers ± 120 μM Spm. **(D)** ^32^P-PA response in *gapc1-1/gapc2-2* and *gapc1-1/gapc2-1* double knock-out mutants respect to wt. Mean ± SD (*n* = 3). All experiments were repeated twice with similar results. Different letters indicate values significantly different according to Student-Newman-Keuls test at *P*-value < 0.05.

Spermine is known to cause an accumulation of NO and H_2_O_2_ (Cona et al., 2006; Tun, 2006; Moschou et al., 2008), which is likely mediated by polyamine oxidase (PAO) and diamine oxidase (DAO) activities (Tun, 2006; Wimalasekera et al., 2011). We confirmed that Spm was able to trigger an increase in H_2_O_2_ and NO under our conditions, as evidenced by the increase in fluorescence of their reporters, i.e., 2′,7′-dichlorofluorescein diacetate (H_2_DCFDA; Figure 6A) and diaminorhodamine-4M acetoxymethyl ester (DAR-4M AM; Figure 6B).

Since PLD activity can be activated by H_2_O_2_ (Wang and Wang, 2001; Zhang et al., 2003, 2009) or by NO (Distéfano et al., 2008; Lanteri et al., 2008; Raho et al., 2011), this could be a potential mechanism by which the production of PA was stimulated, especially since H_2_O_2_ and NO have been found to act upstream of PLDδ in response to ABA induced-stomatal closure (Distéfano et al., 2012). Similarly, H_2_O_2_ has been found to promote the binding of cytosolic glyceraldehyde-3-phosphate dehydrogenase (GAPC) to PLDδ and increase its activity (Guo et al., 2012).

To investigate whether H_2_O_2_ and NO were responsible for the Spm induced-PA response, the effects of ROS scavenger, DMTU and NO scavenger, carboxy-PTIO (cPTIO) were analyzed. While able to significantly reduce the accumulation of Spm-derived H_2_O_2_ and NO (Figures 6A,B), the scavengers had no effect on the Spm induced-PA response (Figure 6C), suggesting that the increase in PA was independent of these secondary metabolites. Moreover, double *gapc1-1 gapc2-1* or *gapc1-1 gapc2-2* knock-out mutants, revealed a PA response similar to wild type (Figure 6D). These results indicate that the Spm-induced PA is not caused via ROS- or NO induction.

Testing polyamines on seedling growth, we observed a significant reduction in primary root growth (Supplementary Figure S3). Previous reports have shown that this root growth inhibition is associated to H_2_O_2_ accumulation, derived from PAO activity (de Agazio et al., 1995; Couée et al., 2004; Tisi et al., 2011). However, loss-of function *pldδ* seedlings did not show any apparent root phenotype when transferred to agar plates containing μM concentrations of Spm. Only a slight increase in root growth inhibition with respect to wt was observed at higher Spm concentrations, i.e., 150 μM (Supplementary Figure S3). Those results are consistent with previous reports indicating that *pldδ* is more sensitive to H_2_O_2_-induced stress (Zhang et al., 2003).

### PLDδ Is Involved in the Spm-Induced K^+^-Efflux Response in the Root-Elongation Zone

**FIGURE 7:**
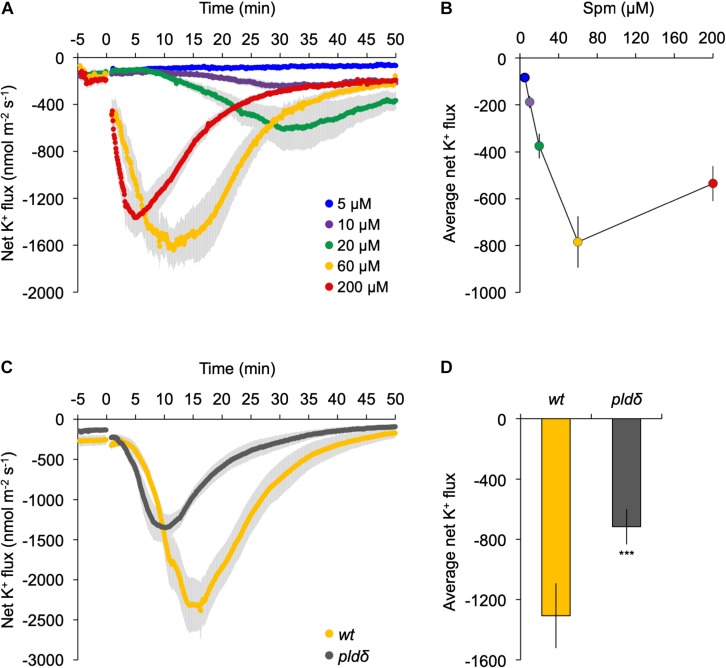
K^+^ flux at the root elongation zone is strongly reduced in *pldδ* mutant. **(A)** Dose-response analyses of K^+^ flux kinetics measured by MIFE in the root tip. Spm was added at the indicated concentrations (*t* = 0). **(B)** Quantification of the average K^+^ flux during the 50 min period of treatment. **(C)** Transient K^+^-flux kinetics in wt- and *pldδ* seedlings upon addition of 60 μM Spm at *t* = 0. **(D)** Quantification of the average K^+^ flux over 30 min of Spm treatment (means ± SE, *n* = 6–7). Asterisks indicate significant differences using Student’s *t*-test: *P < 0.05, **P < 0.01, ***P < 0.005. For all MIFE data, negative values represent net efflux of ions across the PM into the apoplast.

Application of exogenous polyamines has been shown to trigger a K^+^ efflux in pea- (Zepeda-Jazo et al., 2011) and maize roots (Pandolfi et al., 2010), which has consequences for the membrane potential, inducing the plasma membrane to depolarize (Ozawa et al., 2010; Pottosin et al., 2014). To study this in our context, we performed MIFE ion-flux analyses at the root elongation zone of Arabidopsis seedlings using different concentrations of Spm. As shown in Figures 7A,B, a clear dose-dependent efflux of K^+^ was detected, which correlated with the response in PA (Figure 2B). While the Spm induced-K^+^ efflux slowly restored to pre-treatment values after 50 min with 60–200 μM, the efflux persisted when only 10–20 μM Spm was used.

To investigate potential involvement of PLDδ, the response of wild type was compared with that of the *pldδ* knock-out mutant in the same root zone. Prior to Spm application, roots showed a small net K^+^ efflux of 150–250 nmol m^–2^ s^–1^, likely due to transferring seedlings from nutrient-rich MS medium (∼20 mM K^+^) to poorer, basic-salt medium (BSM; 0.2 mM K^+^; Figure 7). Upon 60 μM Spm application to wild-type seedlings, the K^+^ efflux increased gradually, reaching a peak around 15 min, and returning to basal levels after ∼50 min (Figure 7C). In *pldδ*, the response was significantly different, showing an faster K^+^ efflux peak at 10 min, and recovery (Figure 7C). Overall, *pldδ* showed ∼60% reduction in net K^+^ loss compared to wt (Figure 7D), placing PLDδ and derived PA upstream of the Spm induced-K^+^ efflux. Since the Spm induced-K^+^ efflux is completely abolished by gadolinium (Supplementary Figure S4), these results indicate that PLDδ is likely activated by Spm from the inside of the cell, after its uptake.

## Discussion

### Polyamines Trigger a Charge-Dependent PA Response

Polyamines are naturally occurring polycationic molecules involved in a plethora of cellular events (Tiburcio et al., 2014), yet it is still largely unknown how this works at the molecular level. Here, a link to the formation of the lipid second messenger PA is reported, which is predominantly generated by PLDδ and plays a role in Spm induced-K^+^ efflux.

In this paper, we show that low μM concentrations of Spm trigger a rapid (minutes) PA response in the roots of Arabidopsis seedlings (Figure 2). In older plant material, Spm also triggered a PA response in leaves (Supplementary Figure S1), so multiple tissues are sensitive to Spm. Other polyamines, like Spd, Put and Dap were also able to trigger an accumulation in PA but this required higher concentrations, especially the diamines. In contrast, tSpm was as effective as Spm (Figure 1). These results indicate that the capacity of polyamines to activate the formation of PA is charge-dependent, with Spm^4+^ = tSpm^4+^>Spd^3+^>>Put^2+^, which may indicate an electrostatic interaction between the positive charges of the polyamine and a negatively charged target (Bertoluzza et al., 1988; Kurata et al., 2004; DeRouchey et al., 2010; Rudolphi-Skórska et al., 2014).

### Triggering PA Response Requires Uptake of Polyamines

The saturation of the response at relatively low concentrations (Figure 2) may reflect a saturation of polyamine uptake (Pistocchi et al., 1987; Ditomaso et al., 1992a). In that regard, the Spm induced-PA responses were found to be strongly reduced in the plasma membrane localized PUT, *RMV1*-KO, also known as LAT1 or PUT3 (Fujita et al., 2012; Fujita and Shinozaki, 2014), while overexpression of *RMV1* resulted in a much stronger PA response upon Spm treatment (Figure 3). The affinity of this transporter for Spm (*K*_m_ = 0.6 μM), Spd (*K*_m_ = 2.2 μM), and Put (*K*_m_ = 56.5 μM), respectively (Fujita et al., 2012; Fujita and Shinozaki, 2014), is consistent with their potency to activate a PA response and indicates, together with the inhibition of the PA response by gadolinium (Figure 3), that polyamines are taken up before triggering a PA increase.

### Link Between Polyamine Synthesis and PLDδ

Differential ^32^P-labeling experiments combined with transphosphatidylation assays revealed that part of the PA response was generated via the PLD pathway. Using T-DNA insertion *PLD*-KO mutants, we found that the majority of the PA is generated through PLDδ. Arabidopsis contains 3α-, 2β-, 3γ-, 1δ-, 1ε-, and 2ζ PLDs, which differ in amino acid-sequence conservation and lipid-binding domains (Bargmann and Munnik, 2006; Hong et al., 2016; Hou et al., 2016). PLDδ is typically localized in plasma membrane facing the cytosol (Wang and Wang, 2001; Pinosa et al., 2013) while all others PLDs are cytosolic though can transiently bind to various microsomal membranes (Wang, 2002). PLDδ has been implicated in drought and salinity stress (Katagiri et al., 2001; Bargmann et al., 2009; Distéfano et al., 2015) and in freezing tolerance (Li et al., 2004), which are stress responses in which polyamines have also been implicated to play a role (Alcázar et al., 2010). The specific involvement of PLDδ further implies that the polyamine induced-PA response predominantly occurs at the plasma membrane.

Both Spm and tSpm activated PLDδ equally well. In Arabidopsis, Spm is synthesized by spermine synthase (SPMS) while tSpm by ACAULIS5 (Panicot et al., 2002; Kakehi et al., 2008). Both are encoded by single genes, *SPMS* and *ACL5*, which are predominantly expressed in the phloem and xylem, respectively, in both roots and leaves (Supplementary Figure S5; Brady et al., 2007; Winter et al., 2007; Sagor et al., 2011; Yoshimoto et al., 2016). Interestingly, their expression strongly overlaps with that of *PLDδ*, especially *SPMS*, which are both strongly induced upon salt- and osmotic stress (Supplementary Figures S5, S6; Katagiri et al., 2001; Brady et al., 2007; Winter et al., 2007). So potentially, the local synthesis and/or transport of spermine could activate PLDδ to generate PA. In agreement, significantly increased PA levels were found in an SPMS-overexpressor line (35S::SPMS-9; Gonzalez et al., 2011) at control conditions (Supplementary Figure S7A). An *spms-2* KO line exhibited normal PA levels, however, in response to salt stress, a strongly reduced PA response was found while the SPMS-overexpressor line revealed a much higher PA response than wt (Supplementary Figures 7A,B). These results support the idea of a novel, interesting link between PA and Spm in stress responses. Alternatively, since polyamines are rich in soil and produced by various microbes (Young and Chen, 1997; Chibucos and Morris, 2006; Zandonadi et al., 2013; Zhou et al., 2016), our observation that extracellular polyamines trigger intracellular PA responses may also reflect the action of natural, exogenous polyamines.

### PA Function

Phosphatidic acid is an important plant phospholipid. Besides its role as precursor for all glycerolipids at the ER, PA has emerged as important lipid second messenger, generated through the PLC/DGK- and/or PLD pathways in response to various (a)biotic stresses, including plant defense, wounding, salt, drought, cold, and heat stress, where it is linked to various cellular processes, like vesicular trafficking, membrane fission and -fusion, and transport (Munnik et al., 2000; Testerink and Munnik, 2005, 2011; Vergnolle et al., 2005; Zhao, 2015; Hong et al., 2016; Hou et al., 2016). A local accumulation of PA in cellular membranes may affect the enzymatic- or structural properties of protein targets in that membrane or, alternatively, recruit cytosolic protein targets via PA-binding domains. PA targets include protein kinases, phosphatases, ion transporters, PEPC, GAPDH, NADPH oxidases (Rboh) (Kim et al., 2013; McLoughlin et al., 2013; Pleskot et al., 2013; Putta et al., 2016; Ufer et al., 2017). Here, evidence is provided for a role of PA in ion transport. MIFE analyses showed that Spm triggers a rapid efflux of K^+^ ions, which was strongly reduced in the *pldδ* mutant (Figure 7), indicating a direct or indirect role for PA in K^+^ gating. Associated to Spm uptake, we observed a fast net H^+^ influx (cytosolic acidification) followed by a gradual increase of H^+^ efflux (cytosolic alkalinisation), which correlated with the K^+^ efflux peak and its gradual recovery (Supplementary Figure S8). The cytosolic alkalinisation is related to the opening of voltage-gated inward-rectifying K^+^ channels (K_in_) to compensate the K^+^ efflux (Dreyer and Uozumi, 2011; Karnik et al., 2016). In animal cells, PA has been shown to regulate voltage-gated potassium (Kv) channels and has been proposed to stabilize K^+^-inward channels (K_in_) in its closed conformation, thus reducing K^+^ inward currents (Hite et al., 2014). In plants, PA has been shown to inactivate K_in_ channels in guard cells of *Vicia faba* and Arabidopsis (Jacob et al., 1999; Uraji et al., 2012), in which PLDδ has been implicated (Uraji et al., 2012). These observations are in agreement with a role for PA in regulating K^+^ fluxes of which the precise mechanism requires further investigation.

In summary, we provide molecular evidence that polyamines functionally require PLD and PA for their mode of action. This knowledge and the use of PLD- and polyamine synthesis mutants may shed new light on this phenomenon in other studies.

## Data Availability

All datasets for this study are included in the manuscript and the Supplementary Files.

## Author Contributions

XZ, SS, and TM designed the experiments. LS performed the MIFE experiments while XZ performed the rest. MF, AT, and MH added materials, ideas and discussions. XZ and TM wrote the manuscript.

## Conflict of Interest Statement

The authors declare that the research was conducted in the absence of any commercial or financial relationships that could be construed as a potential conflict of interest.
