# Supplementary material for: Extracellular Spermine Triggers a Rapid Intracellular Phosphatidic Acid Response in Arabidopsis, Involving PLDδ Activation and Stimulating Ion Flux
Source: Front Plant Sci. 2019 May 21;10:601. doi: 10.3389/fpls.2019.00601 (PMC6537886; doi:10.3389/fpls.2019.00601)

SUPPLEMENTARY MATERIAL

Zarza et al., 2019

Supplementary Figure S1 | Spm induced-^{32}P-PA response in Arabidopsis mature leaves.
Leaf disks from 3-weeks-old Arabidopsis plants were labelled O/N and the treated for 30 min at indicated concentrations of Spm. Lipids were subsequently extracted and the levels of ^{32}P-PA quantified. Data are the mean ± SD of three independent experiments (n = 9).

Supplementary Figure S2 | PA response in LAT knock-out mutants.
Spm induced-PA responses in L-type amino acid transporter (LAT) quadruple knock-out lines *lat1/2/3/5* and *lat1/2/4/5*. Seedlings were ^{32}P-labelled O/N and the next day treated for 30 min with or without 60 µM Spm and the PA response calculated.

Supplementary Figure S3 | Effect of Spm on root growth of Arabidopsis seedlings.
(**A**) Inhibitory effect of root growth in Arabidopsis seedlings grown for 9 days on plates with increasing concentrations of Spm. (**B**) Comparison between wild-type and *pldδ* seedlings grown for 5 days on plates containing standard medium and then transferred to plates with or without 150 µM Spm. The image was taken 4 days after transfer (DAT). White dashes indicate the position of the root tip when the seedlings were transferred. (**C**) Quantification of the growth ratio of the main root (MR) at 4 DAT in response to 0, 60 and 150 µM Spm. Five independent plates per treatment were used, containing eight seedlings each. All experiments were repeated twice with similar results.

Supplementary Figure S4 | Gadolinium blocks Spm induced-K^+ flux.
Quantification of the average K^+ flux measured over 30 min in 5 days-old seedlings pre-treated for 60 min with or without 100 µM $GdCl_3$ prior to 60 µM Spm application (t = 0). Mean ± SD (n = 6 - 7). Negative values represent net efflux of ions to the apoplast.

Supplementary Figure S5 | Expression of *SPMS*, *ACL5* and *PLDδ* in roots.
Comparison of cell- and tissue-specific expression between *SPMS* (**A**), *ACL5* (**B**) and *PLDδ* (**C**) as indicated by the Arabidopsis eFP Browser (Brady et al., 2007; Winter et al., 2007).

Supplementary Figure S6 | *SPMS*-, *ACL5*- and *PLDδ* expression in response to abiotic stress.
Comparison tissue-specific expression between *SPMS* (**A**), *ACL5* (**B**) and *PLDδ* (**C**) in response to several abiotic stresses as predicted by the Arabidopsis eFP Browser (Kilian et al., 2007; Winter et al., 2007; Dinneny et al., 2008).

Supplementary Figure S7 | Salt stress-induced PA responses are stronger in an SPMS-overexpressor line and smaller in an *spms* KO- mutant.
NaCl induced-PA responses in (**A**) *Pro35S::SPMS-9* and (**B**) *spms-2* KO lines. Seedlings were ^{32}P-labelled O/N and the next day treated with or without 500 mM NaCl for 30 min.

Supplementary Figure S8 | H^+ flux kinetics in seedlings treated with Spm
(**A**) Dose-response analyses of H^+ flux kinetics measured by MIFE at the elongation zone of the root tip of 5 days-old Arabidopsis wt seedlings. Spm was added at indicated concentrations at t=0. Six to seven seedlings were analysed per treatment. (**B**) Correlation of the dose-response experiments between time-to-peak for influx H^+ and time-to-peak for efflux K^+. For all MIFE data, negative values represent a net efflux of ions to the apoplast.

Supplementary Figure S1 – Zarza *et al*.

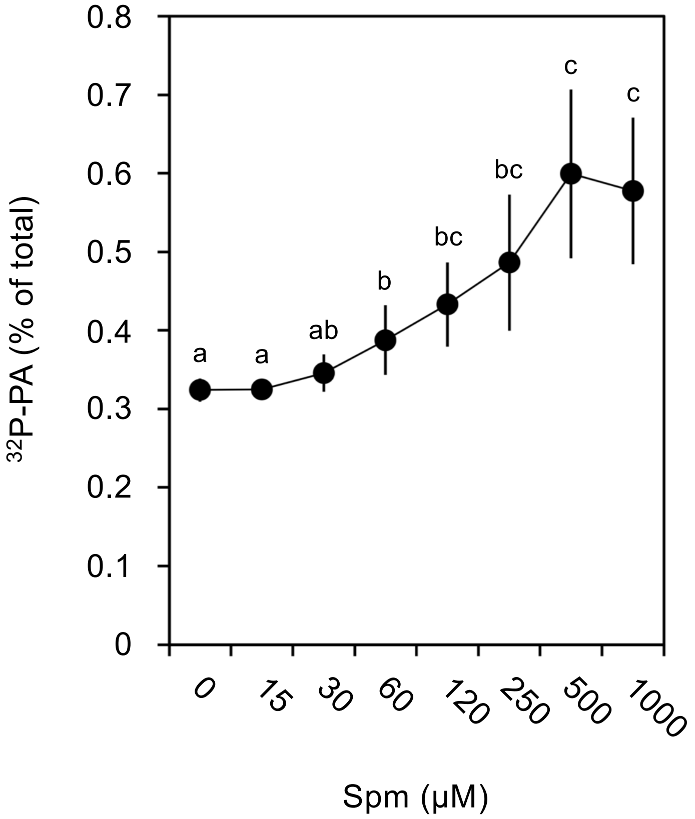

Supplementary Figure S2 – Zarza *et al*.

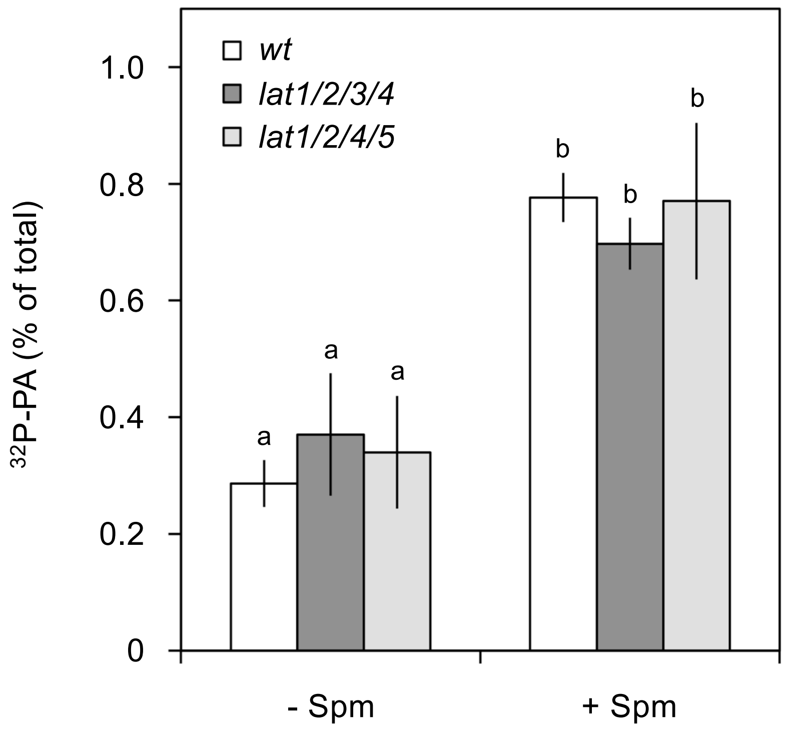

Supplementary Figure S3 – Zarza *et al*.

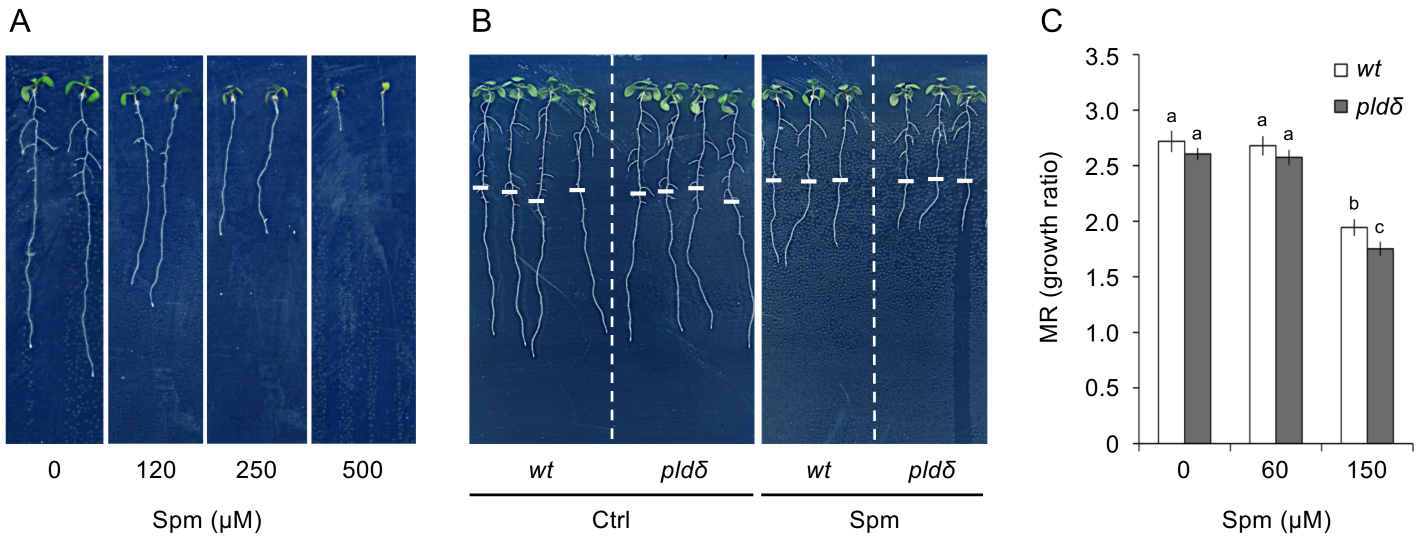

Supplementary Figure S4 – Zarza *et al*.

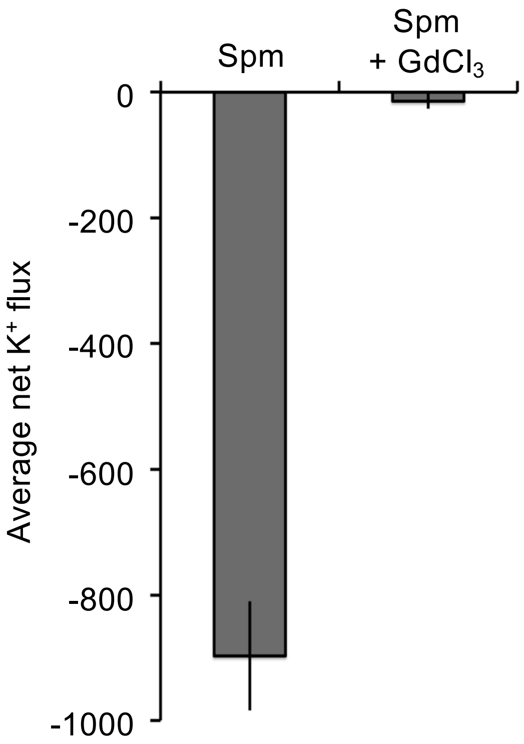

Supplementary Figure S5 – Zarza *et al*.

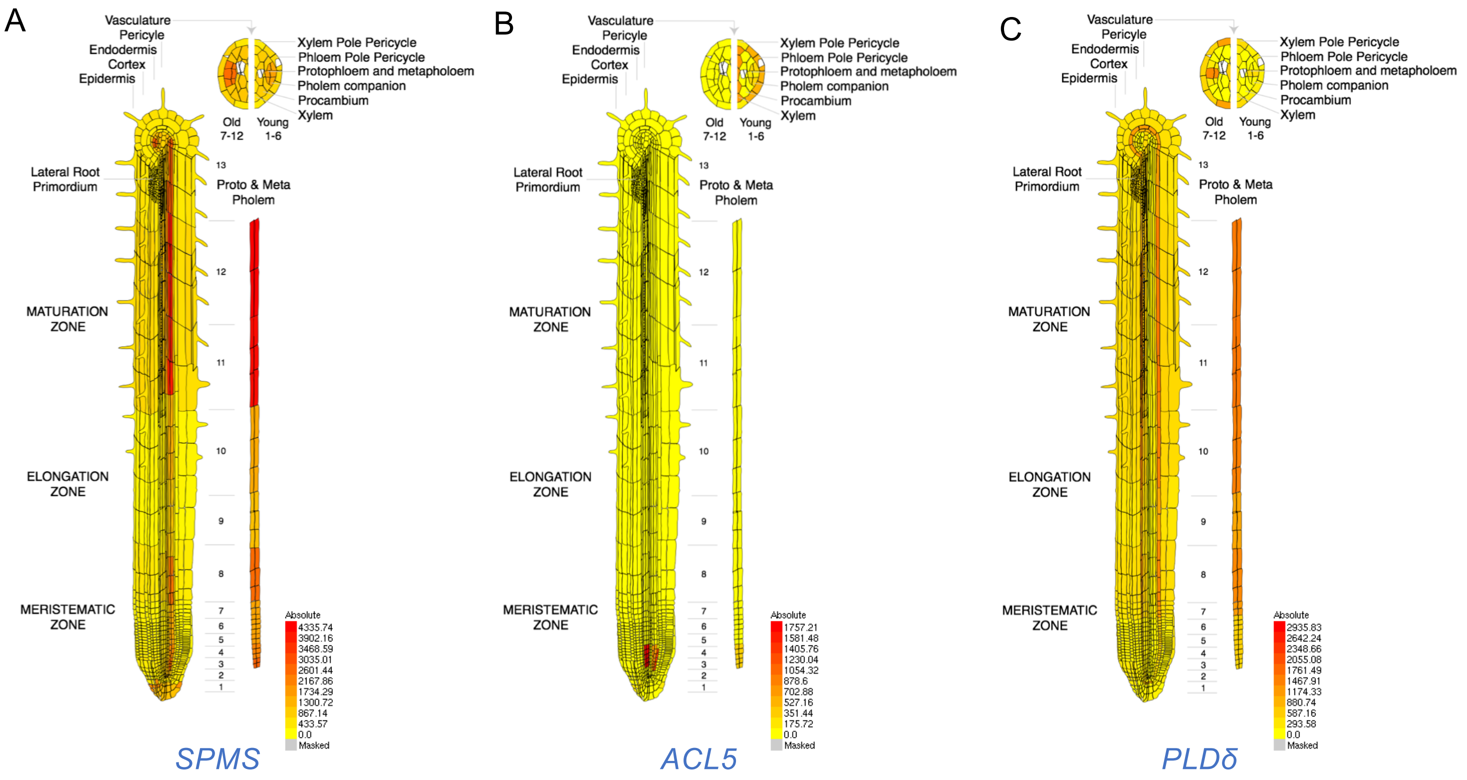

Supplementary Figure S6 – Zarza *et al*.

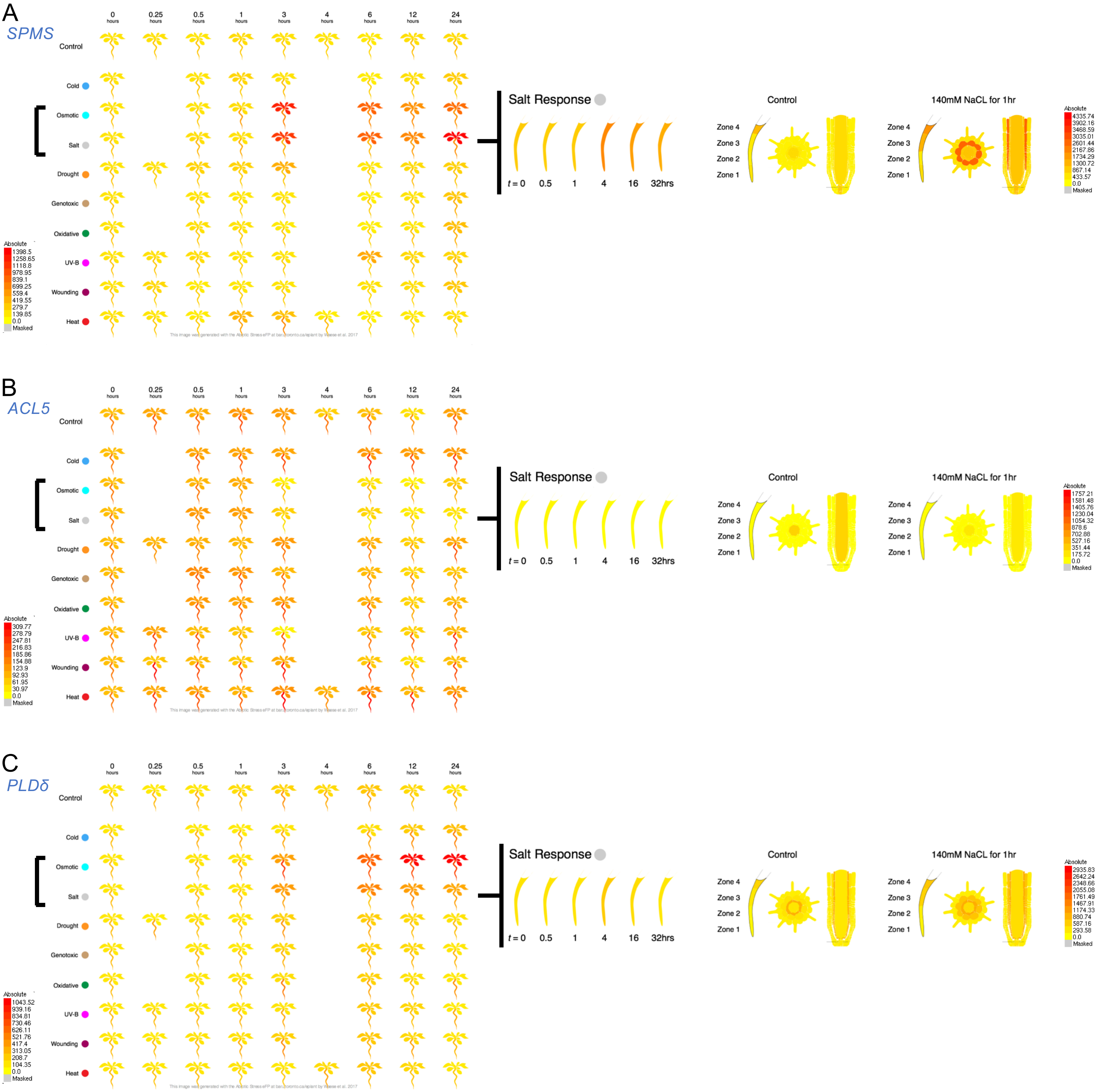

Supplementary Figure S7 – Zarza *et al*.

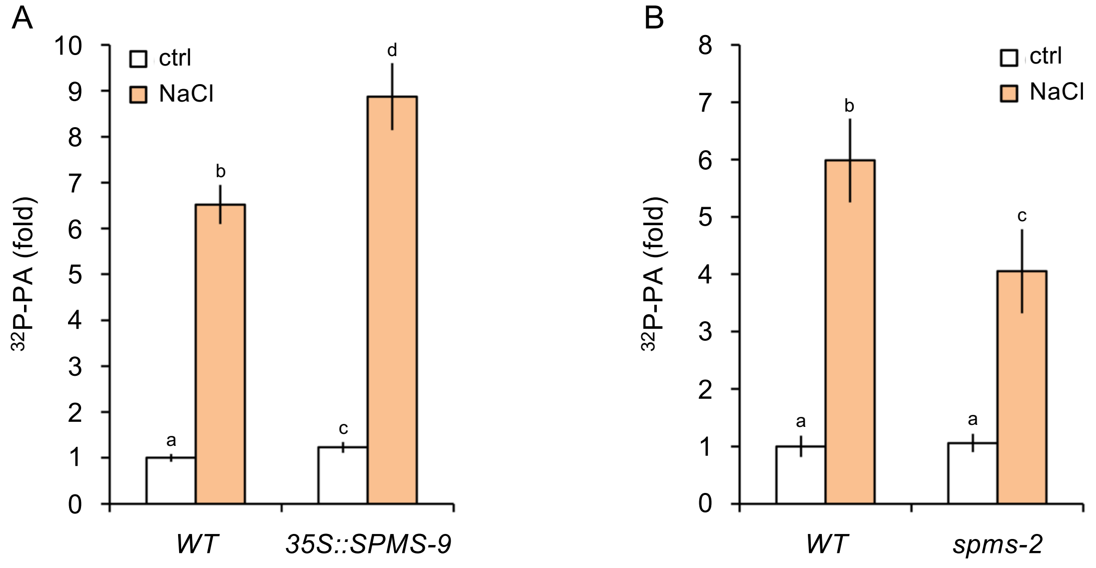

Supplementary Figure S8 – Zarza *et al*.

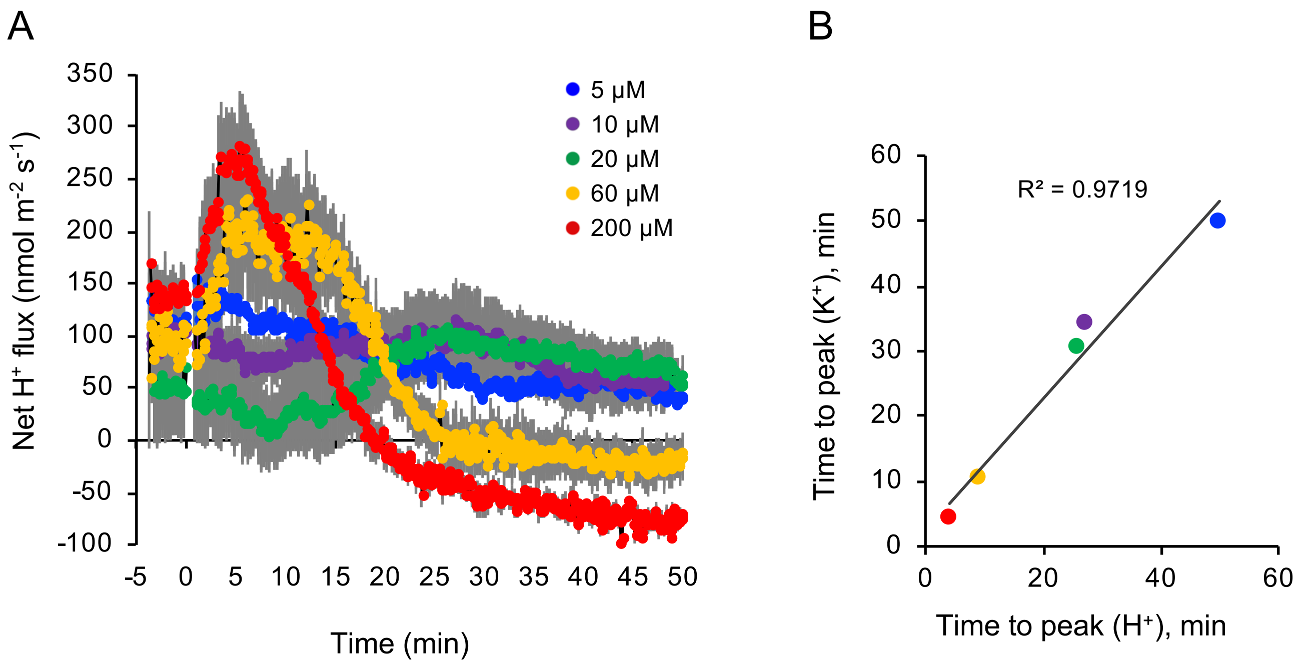

Supplement: Supplementary file 1 [file Presentation_1.pdf]
